# Hydration Kinetics of Portland Cement–Silica Fume Binary System at Low Temperature

**DOI:** 10.3390/ma12233896

**Published:** 2019-11-26

**Authors:** Yao Li, Yonggang Deng, Runqing Liu

**Affiliations:** School of Materials Science and Engineering, Shenyang Ligong University; Shenyang 110168, China

**Keywords:** cementitious material, hydration kinetics, low temperature, hydration model

## Abstract

Portland cement–silica fume binary cementitious materials are widely used in engineering construction and have been investigated from micro- to macroscopic aspects. However, the theoretical background on the hydration kinetics of the binary system has not been sufficiently covered in the literature. In this study, the hydration dynamic characteristics of the Portland cement–silica fume binary system curing at low temperature were investigated. Hydration kinetics equations were optimized and a hydration model followed by a computer program was developed to calculate the reaction rate constant K and the reaction order *n*. This model presented that the hydration process of the binary system at low temperature could be divided into three stages, namely, nucleation and growth (NG), interactions at phase boundaries (I), and diffusion (D). The *n* values for the binary system varied in the range of 1.2 to 1.6, indicating that the hydration of the binary system at low temperature was a complex elementary reaction. Silica fume can reduce the total heat at the later stage of the hydration and the reaction order *n*, but increase the heat flow at the accelerating stage and the hydration rate constant K. Low temperature prolonged the hydration induction period, decreased and delayed the secondary exothermic peak, as well as reduced the *n* and K value.

## 1. Introduction

Winter concrete construction projects demand more strict technical standards, and the construction process needs to be improved and updated. To fully understand the hydration mechanism of the cementing material at low temperature in concrete, its hydration properties demand further investigation, which could provide a theoretical foundation for protecting the concrete freezing damage from low temperature. The macro- and micro-characteristics of concrete are affected by the hydration of the cementing materials [1,2,3]. In general, the hydration process of cementing materials can be divided into hydration products, hydration rate, and hydration degree [1,4]. These three aspects could be characterized in terms of the kinetics and thermodynamics of the hydration process [5,6].

Ba et al. [7] reported the hydrodynamic parameters of cement materials at low temperature, and indicated that the formation of the internal structures provide a further driving force for the hydration. Yan et al. [8] researched the hydration process of Portland cement hydrated at low temperature, from the aspects of mechanical properties, hydration products, and microstructures. He et al. [9] proposed a hydration heat model at low temperature from the point of physicochemical and thermodynamic using semiconductor technology.

The models proposed by the researchers not only include hydrodynamic models concerning a single mineral, but the hydration models of different types of cement and cementing materials [10,11,12,13,14]. Krstulovic and Dabic [15] provided a hydrodynamic model, which was based on three basic processes: Nucleation and growth (NG), interactions at phase boundaries (I), and diffusion (D). The authors pointed out that the slowest one dominates the hydration process as a whole, and thus it was of significance to determine the hydration rates of the three processes for hydration of cement. Yan et al. [16] fitted the data of hydration heat by thermal spectrum reconstruction and characterized the three processes using integration methods and differential equations. Thereafter, the kinetics of the hydration model with regards to the reaction rate constant K, reaction order *n*, and apparent activation energy E_a_ were thus calculated.

Few available studies illustrate the hydrodynamics equations considering the dynamic factors with regards to low temperature curing for the composite cementing system. On the other hand, the Krstulovic–Dabic model could be applied to a wide range of cementing systems and not confine to mono cement to composite cementing systems. Furthermore, the extension of the model can be achieved by the use of mathematical models in terms of the factors that affect the hydration. Thus, the Krstulovic–Dabic was selected to fit the hydration kinetics model of the Portland cement–silica fume composite cured at low temperature in which the Portland cement, silica fume, and sodium nitrite were chosen as the basic components. In addition, the experiment was carried out at different temperatures varying from of −10 °C to 5 °C and aimed to study the effect of silica fume and low temperature on the heat flow and heat release of the composite. Further, the reaction rate K and reaction order *n* were calculated and thus the primary factors that control the hydration process of the composite were determined in terms of low temperature and silica fume. Finally, the hydration kinetics model of the Portland cement–silica fume cementing material under low temperature condition was established using regression analysis. The hydration kinetics model can provide a powerful method to simulate the hydration degree of the Portland cement–silica fume cementing material. Especially, the obtained parameters from the model specifically reflected the hydration stages of cementitious cement at low temperature, which could lay a theoretical foundation for the hydration research of low temperature concrete and thus give a guidance for winter concrete construction projects.

## 2. Materials and Methods

### 2.1. Materials

Ordinary Portland cement (P II 52.5), produced by Onota Co. Ltd (Dalian, China) was used in the experiments. Silica fume with a specific gravity of 2.20 and special surface area of 22,500 m^2^/kg was employed. The chemical compositions of cement and silica fume are listed in Table 1 and Table 2. Standard sand (ISO) was used as fine aggregate. Other commonly used materials, such as water and anti-freezer (sodium nitrite), are based on GB51081-2015. The mix proportion applied in this study is given in Table 3. The water-to-binder (the sum of cement and silica fume) ratio used for all mixtures is 0.42 and contain silica fume in the amount from 0% to 12% by weight of binder.

### 2.2. Test Methods

In the hydration heat test, a low temperature thermoactive microcalorimeter was used. The data of hydration heat and rate were collected at different temperatures and time. Finally, the hydration kinetics model of the Portland cement–silica fume cementing material at low temperature was established and model coefficients were revised using regression analysis.

### 2.3. Hydration Kinetics Model Based on Krstulovic–Dabic Equation

According to Krstulovic–Dabic model, microcalorimetry is characterized by continuous determination of heat released during hydration of cement constituents. By means of the heat released, it is possible to determine the hydration degree relative to hydration duration according to the Equation (1).

(1) $$\alpha \left(t\right)=\frac{Q\left(t\right)}{Q_{max}},$$

where *Q(t)* is the heat released by time t, and Q_max_ represents the total heat that a sample can release. For pure minerals, *α(t)* is the mineral reaction degree, while for cement it is a consequence of the combined effect of heat released by all constituents present. *Q(t)* in here could be calculated from data gathered by test set-up. *Q_max_* was obtained by data fitting and calibration, the principle is described in detail in References [17,18,19].

In this article, we took the data of cementitious material with 10% silica fume, 5 °C reaction condition, and 180 h hydration time as an example to calculate the degree and rate of hydration. First, in order to determine *Q_max_*, the heat output curve was linearly fitted by hydration kinetics model using the data generated during 165–175 h in which *1/t* in accordance with the abscissa, 1/*Q* is the ordinate and obtained Equation (2):

$$1/Q=0.01781\left(1/t\right)+3.53408\times 10^{-4},$$

(2) $$\mathrm{sd}=1.57726\times 10^{-8},r=0.99996.$$

Apparently as *t* approaches infinity, *1/t* approaches zero and 1/*Q* is replaced by 1/*Q_max_*. Therefore, *Q_max_* calculated by Equation (2) in the present study is 2829.59073 J.

It is known that the hydration heat from the first exothermic peak to the end of induction period is about 5% of total quantum of heat and can be ignored. In the present test, the specimens were removed from the refrigerator and then placed in a hydration heat tester, the process led to the closing of induction period. Consequently, in solving kinetics model, the time before the ending of the induction period needs to be removed and the equations of nucleation and crystal growth (NG), interactions at phase boundaries (I), and diffusion (D) in Krstulovic–Dabic model can be converted into Equations (3)–(5):

(3) $${\left[-\ln\left(1-\alpha \right)\right]}^{1/n}=K_{NG}\left(t-t_{0}\right)=K_{NG}^{\prime }\left(t-t_{0}\right),$$

(4) $${\left[1-{\left(1-\alpha \right)}^{1/3}\right]}^{1}=K_{I}r^{-1}\left(t-t_{0}\right)=K_{I}^{\prime }\left(t-t_{0}\right),$$

(5) $${\left[1-{\left(1-\alpha \right)}^{1/3}\right]}^{2}=K_{D}r^{-2}\left(t-t_{0}\right)=K_{D}^{\prime }\left(t-t_{0}\right).$$

Equations (3)–(5) can also be expressed as Equations (6)–(8):

(6) $$\ln\left[-\ln\left(1-\alpha \right)\right]=n\ln K_{NG}^{\prime }+n\ln\left(t-t_{0}\right),$$

(7) $$\ln\left[1-{\left(1-\alpha \right)}^{1/3}\right]=\ln K_{I}^{\prime }+\ln\left(t-t_{0}\right),$$

(8) $$2\ln\left[1-{\left(1-\alpha \right)}^{1/3}\right]=\ln K_{D}^{\prime }+\ln\left(t-t_{0}\right).$$

The above equations after differentiation with respect to *t* are as Equations (9)–(11):

(9) $$\left[\frac{d\alpha }{dt}\right]=K_{1}\left(\alpha \right)=K_{NG}^{\prime }n\left(1-\alpha \right){\left[-\ln\left(1-\alpha \right)\right]}^{\left(n-1\right)/n},$$

(10) $$\left[\frac{d\alpha }{dt}\right]=K_{2}\left(\alpha \right)=K_{I}^{\prime }{\left(1-\alpha \right)}^{2/3},$$

(11) $$\left[\frac{d\alpha }{dt}=K_{3}\left(\alpha \right)=\frac{K_{D}^{\prime }3{\left(1-\alpha \right)}^{2/3}}{2-2{\left(1-\alpha \right)}^{1/3}}\right].$$

Equations (9)–(11) are expressions for the hydration rate and it is independent of *t_0_.*

Using Equations (2)–(4), the reaction order and rate constant are determined according to linear fitting. To compare with the experimental result, we took *sd* assessment methods of evaluate indexes to fit NG, I, and D data for each phase. Finally, the optimum hydration kinetics model was created.

## 3. Results and Discussion

### 3.1. Hydration Heat Analysis of Portland Cement–Silica Fume Binary System

#### 3.1.1. Effect of Silica Fume on the Hydration Heat and Hydration Rate

**Figure 1:**
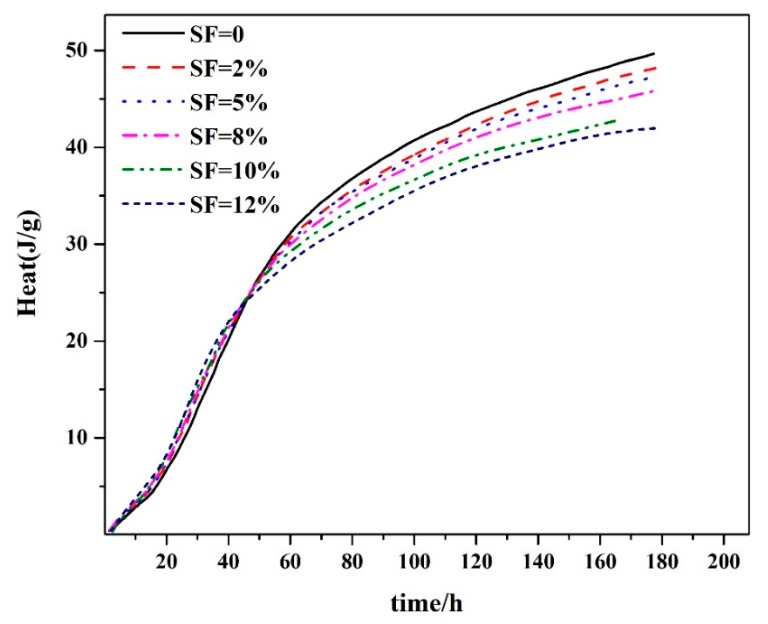
Heat of the composite cementitious system.

**Figure 2:**
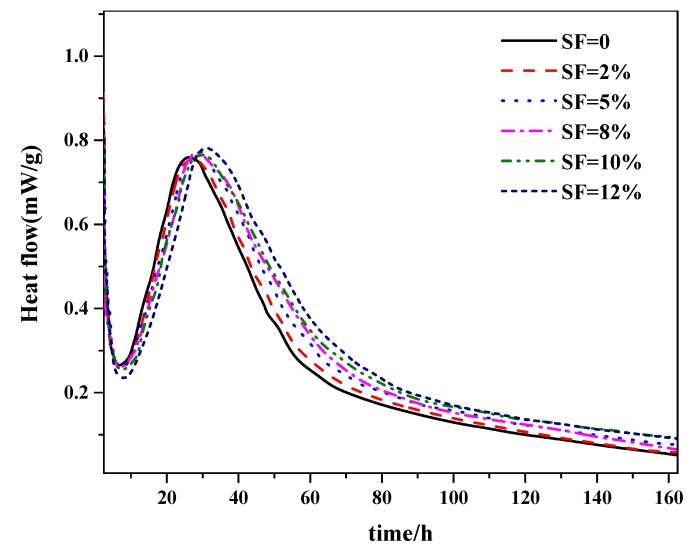
Heat flow of the composite cementitious system.

The hydration heat and rate curves of composite cementitious materials are presented in Figure 1 and Figure 2, respectively (experiment temperature is 5 °C, sodium nitrite is 8.5%, silica fume volume fractions were 0%, 2%, 5%, 8%, 10%, and 12%). We can see a trend that hydration heat decreases with increase in the fraction of silica fume for all the materials, but the differences are not serious (Figure 1).

It can be seen from Figure 2 that the curves of hydration rate changed significantly when the composite included silica fume, i.e., the hydration induction period was shortened and the exothermic rate was increased during the acceleration period.

In addition, test results indicate that the cement–silica fume binary system can short the dormant period, increase the exothermic rate during the acceleration period, decrease the second hydration heat evolution peak, and reduce the hydration rate during the deceleration period. Moreover, it is becoming clear with the increase of the amount of silica fume, when compared with the hydration heat evolution process of pure cement. 

The mechanism of the phenomenon is explained as follows: During the early stage of hydration, silica fume was hydrolyzed and formed negatively charged SiO^−^, which would combine with Ca^2+^ and alkaline ion to form calcium silicate hydrates (C–S–H). Moreover, as an active filler, silica fume provides an increased nucleation density which would shorten the duration of its induction period. During the acceleration period, silica fume in the cementitious materials can promote cement hydration, but in the later age (deceleration period in which the process of hydration reaction is mainly due to diffusion), hydration is delayed and ion diffusibility is weakened because of the present of a thicker hydration products layer located on the surface of unhydrated cement. This led to a reduction of hydration rate and heat. However, during the later hydration age, the heat of hydration and its rate decreased remarkably when the volume fraction of silica fume exceeded 10%, resulting in a poor mechanical properties and compactness of concrete.

#### 3.1.2. Effect of Temperature on the Hydration Heat and Rate

**Figure 3:**
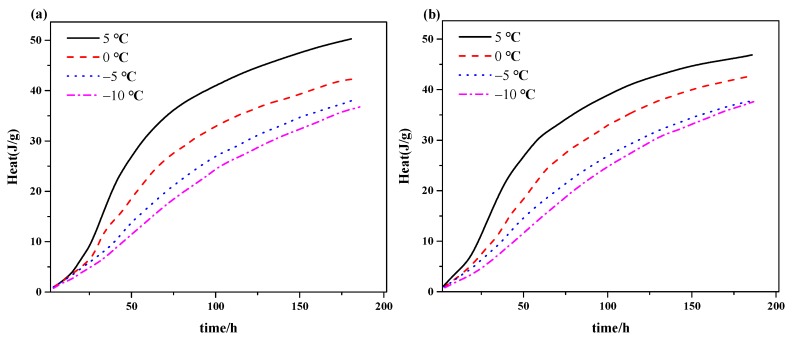
Accumulation of hydration heat curves of the composite cementitious system at different temperatures: (**a**) Cement with no additive and (**b**) mixed with 8% silica fume.

**Figure 4:**
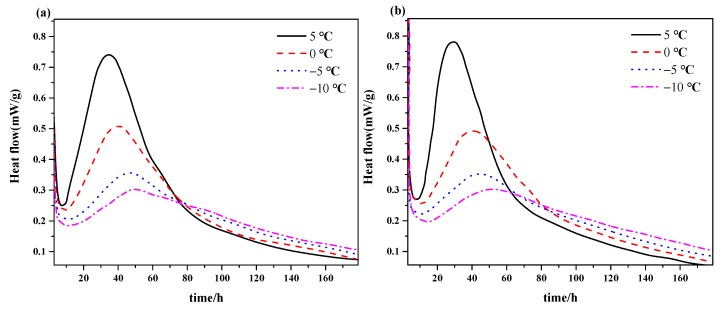
Curves of rate of hydration heat of the composite cementitious system at different temperatures: (**a**) Cement with no additive and (**b**) mixed with 8% silica fume.

The results from the tests of the different temperature (−10 °C, −5 °C, 0 °C, 5 °C) and silica fume content (0%, 8%) are shown in Figure 3 and Figure 4. As observed, the hydration heat was decreased by decrease of reaction temperature from 5°C to −10°C, but the reduction coefficient gradually decreases with the decrease of temperature. Additionally, the hydration heat approached gradually along with the time. In Figure 4, the hydration rate for the composite made with and without silica fume were presented. It can be seen that with a decrease of reaction temperature, the induction period of hydration for all the materials was elongated, the hydration exothermic rate was lowered, and the secondary exothermic peak was decreased and delayed. This trend becomes gradually insignificant with the reduction of reaction temperature. It can be seen from Figure 4, a reduction of secondary exothermic peak of 0.3 mW/g was brought when the temperature was decreased from 5 °C to 0 °C, while 0.02 mW/g for −5 °C to −10 °C. It is known that silica fume can accelerate the hydration process (Figure 3 and Figure 4), but the influence is less comparable with pure cement, especially for the lower temperature.

The above experimental results show that decreasing the temperature can significantly suppress the hydration process, but its decreasing range was smaller with a decreasing reaction temperature. The conclusion was consistent with Van’t Hoff’s equation (the rate of reaction would be increased three to four times with every increase of 10 K) and the macroscopic properties that were obtained from the above results. Moreover, silica fume activity declined with decreasing treated temperature, which would delay the hydration reaction and led to the above phenomenon.

### 3.2. Hydration Kinetics Model of Composition Cementing Material at Low Temperature

#### 3.2.1. Analyze the Model Calculation’s Result Based on Krstulovic–Dabic Equation

According to the heat released data Q(t) and the value of Q_max_, a(t) is calculated first, and then a linear regression in which ln(t-t_0_) in accordance with the abscissa, ln(-ln(1-α)) is the ordinate was applied according to the Equation (6). Finally, the linear fitting equation was obtained:

$$\ln\left[-\ln\left(1-\alpha \right)\right]=1.48036\ln\left(t-t_{0}\right)-5.92513\;\;,$$

(12) sd=0.003, r=0.99989.

The slope and intercept of the fitted line was 1.48036 and 5.92513, which is equivalent to the reaction order *n* and $n\ln K_{NG}^{\prime }$ in Equation (6), thus, the reaction rate constant $K_{NG}^{\prime }$ was 0.01827.

Similarly, based on Equations (7) and (8), two other linear fitting equations were established and the reaction rate constant during stage I and D were 0.00435($K_{I}^{\prime }$) and 0.001($K_{D}^{\prime }$), respectively.

$$\ln\left[1-{\left(1-\alpha \right)}^{\frac{1}{3}}\right]=\ln\left(t-t_{0}\right)-5.43758,$$

(13) sd=0.00702, r=0.99976,

$$2\ln\left[1-{\left(1-\alpha \right)}^{\frac{1}{3}}\right]=\ln\left(t-t_{0}\right)-6.90776,$$

(14) sd=0.00147, r=0.99999.

**Figure 5:**
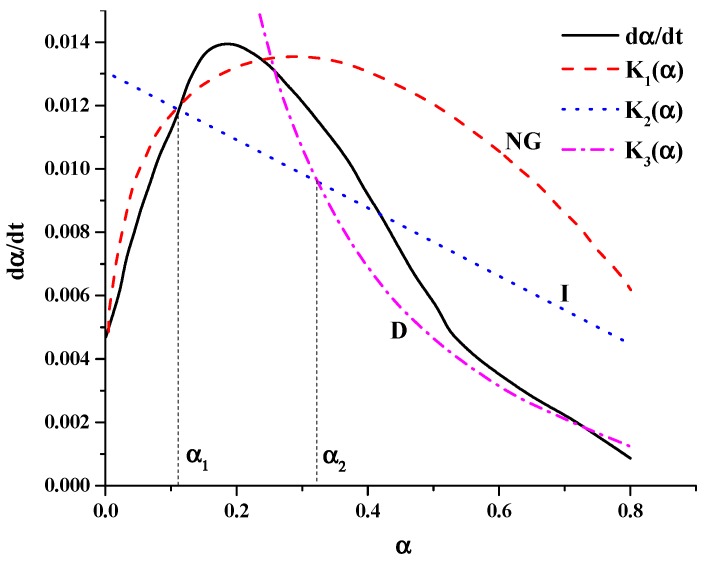
Curves of reaction of hydration of the composite cementitious system at lower temperatures.

According to Equations (9)–(11), the fitting Equations K_1_(α), K_2_(α), K_3_(α) of hydration rate were calculated. The relationship between hydration degree (α) and hydration rate (dα/dt) were fitted as shown in Figure 5. The results show that the fitting results of stage NG and D are better than stage I. The intersection points between K_1_(α) and K_2_(α), K_2_(α) and K_3_(α) are α_1_ and α_2_, respectively (α_1_ and α_2_ are the transformation points from stage NG to I and stage I to D).

It can be seen from Figure 5 that before a_1_ the fitting effect of NG stage is excellent, which indicate nucleation and crystal growth are the main factors to control the process. In the same way, after a_2_, stage D is control by diffusion reaction. However, a bad fitting effect of stage I is observed between α_1_ and α_2_, which agrees well with the fitting result of hydration kinetic model at ambient temperature. This can be interpreted by a weak phase-side reaction (the main control factor during stage I) and a strong nucleation, diffusion processing.

According to the model of Krstulovic–Dabic, hydration kinetics equations of the Portland cement–silica fume binary system at a lower temperature were calculated. The values of n, $K_{\mathrm{NG}}^{\prime }$, $K_{I}^{\prime }$, $K_{D}^{\prime }$ and the intersection points a_1_ and a_2_ were obtained. With this method, we can get the model coefficients at different hydration temperatures.

#### 3.2.2. Effect of Silica Fume on the Hydration Kinetics Evolution

**Figure 6:**
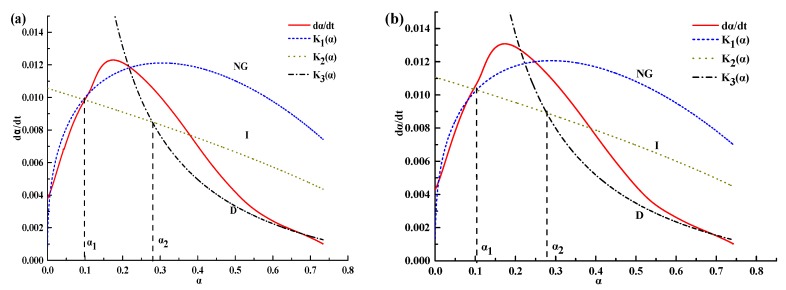

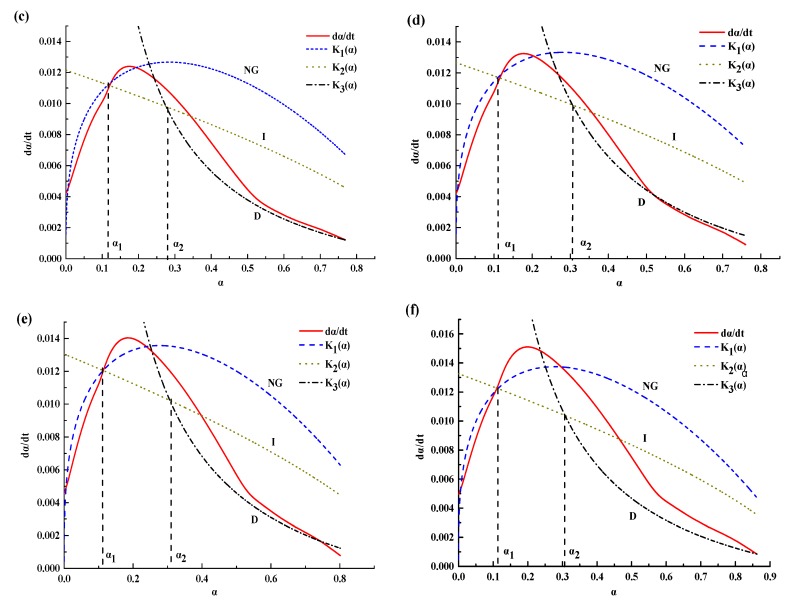
Fitting curves of the composite cementitious system. (**a**) Sample without admixture, (**b**) Sample mixed with 2% silica fume, (**c**) Mixed with 5% silica fume, (**d**) Mixed with 8% silica fume, (**e**) Mixed with 10% silica fume and (**f**) Mixed with 12% silica fume.

Table 4 present the hydration kinetic parameters of composite cementitious incorporating different volume fraction of silica fume and which hydration temperature is 5 °C. According to the calculated results, the curves of hydration rate were fitted (Figure 6). It can be seen that the law of all the fitting results are consistent with Figure 5 and interpreted by different controlling factors of hydration reaction during stage NG, I, and D.

Reaction order *n* during NG stage is summarized in Table 4. We can see that the value of *n* is located in 1.48–1.57, non-integer, which indicates that the hydration reaction of the cement–silica fume binary system at low temperature is nonelementary. Moreover, *n* decreases with increase in the fraction of silica fume. This can be interpreted by the point of physical chemistry: Solution concentration of composite cementitious increases with increase in the fraction of silica fume, and result in the decreases of the reaction rate of nucleation and growth. An interesting aspect is that *n* does not change much with the increase of silica fume content during the binary system (the maximum difference of 0.04 and the minimum difference of 0). 

Furthermore, We can see a trend that $K_{NG}^{\prime }$, $K_{I}^{\prime }$, $K_{D}^{\prime }$ during stage NG, I, and D increase with increase in silica fume content. This is consistent with the fact that silica fume promotes hydration reaction. When the volume fraction of silica fume increased more than 10%, *K* showed a slower and slower rising trend, because the inhibition of an excessive dose of silica fume. On the basis of mechanical properties results we suggest that the best volume fraction is about 8%.

#### 3.2.3. Effect of Temperature on the Hydration Kinetics Evolution

**Figure 7:**
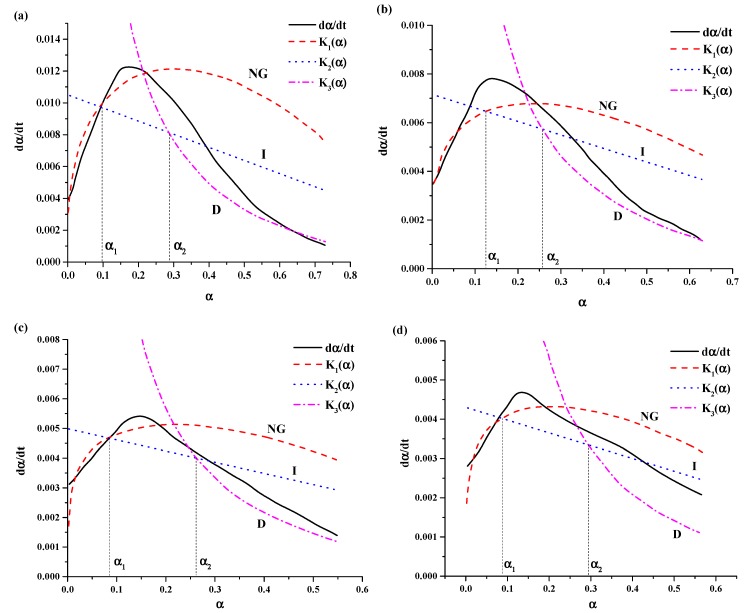
Fitting curves of hydration rate of the samples without admixture at different temperatures: (**a**) 5 °C, (**b**) 0 °C, (**c**) −5 °C and (**d**).−10 °C.

**Figure 8:**
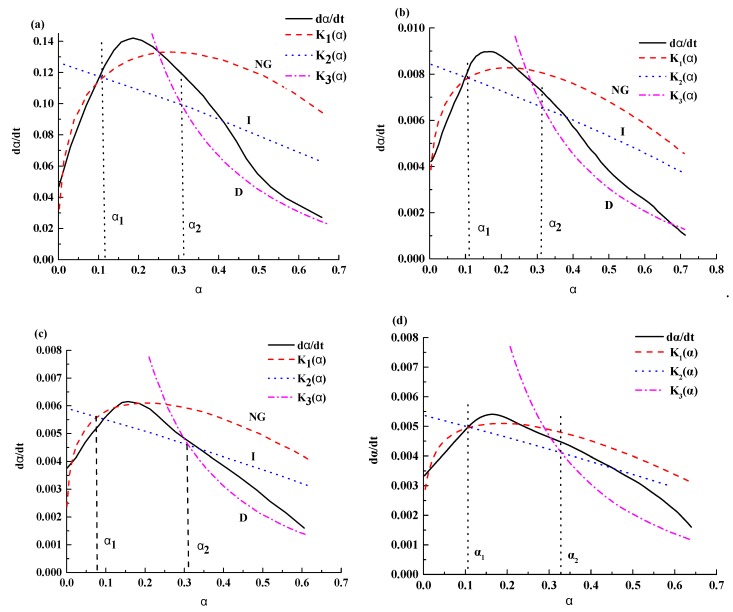
Fitting curves of hydration rate of the composite cementitious system mixed with 8% silica fume at different temperatures. (**a**) 5 °C, (**b**) 0 °C, (**c**) −5 °C and (**d**) −10 °C

Table 5 present the hydration kinetic parameters of composite cementitious incorporating different silica fume and hydration at different temperatures. According to the calculated results, the curves of the hydration rate were fitted (Figure 7 and Figure 8). 

Again, the hydration reaction of a cement–silica fume binary system is nonelementary. The reaction order *n* decreases with decrease in the hydration temperature regardless of pure-cement or binary system. However, with decreasing hydration temperature, *n* decreased slowly (the difference of 0.17 from 5 °C to 0 °C, whereas 0.05 from −5 °C to −10 °C). This could be also attributed to the decreases of the reaction rate during nucleation and growth stages with reducing hydration temperatures. By comparing the values of *n* in type I and type II, we know that *n* decreases with the increase in silica fume. The difference of *n* between type I and type II increases with the decrease of hydration temperature. 

Reaction rates of constant *K* are summarized in Table 5. We can see a trend that *K’_NG_, K’_I_*, and *K’_D_* decreases with decrease in hydration temperature from 5 °C to −10 °C. This means decreased hydration temperature could suppress the hydration reaction, the lower the temperature, the slower the rate of hydration reaction.

By comparing the values of *K* in type I and type II, we know that *K* increases with the increase in silica fume. This indicates that mixed with silica fume in cement, it can promote the hydration reaction rate. However, with temperature decreasing, its proliferation weakened. *K* in lower temperature reduced by 1–2 orders of magnitude compared with *K* in room temperature. The results showed that low temperature had obvious inhibition on the hydration reaction.

In addition, there are no significant differences between Figure 6, Figure 7 and Figure 8. It means that the effect of temperature on the hydration reaction would not influence radically. While the hydration rate was reduced, controlling factors during the transitional stage were weakened and led to a severe deviation occurs.

The hydration kinetics model can effectively simulate the hydration degree of the Portland cement–silica fume cementing material. The obtained parameters from the model specifically reflected the influence of temperature and silica fume on the three hydration stages of cementitious cement. According to the calculation results, the incorporation of silica fume, which could act as nucleating agent, can greatly accelerate the hydration rates of corresponding reaction stages and therefore the construction technical problems in winter can be effectively resolved. 

With regards to the hydration kinetics model, lower *K’_NG_* indicates that the crystal growth stage is the principal factor to control the hydration process of cementitious materials. In other words, in terms of cementitious materials with lower *K’_NG_*, construction projects can add nucleating agents or adopt other methods to accelerate the hydration rates of the cementitious materials. Similarly, lower *K’_I_* represents that interaction between phases in cementitious materials is the principal factor to control the hydration process of cementitious materials. Consequently, making efforts to strengthen the interaction between phases in cementitious materials could accelerate the hydration rate of the cementitious materials. Alternatively, in terms of lower *K’_D_*, which indicates controllable hydration process by diffusion stage, to promote the formation of hydration products, a proper condition for diffusion is required.

## 4. Conclusions

In the present study, the hydration kinetics equations of cement–silica fume binary system under low temperature conditions were optimized and a hydration model followed by a computer program was developed to calculate the reaction rate constant *K* and the reaction order *n*. The main conclusions are as follows:

(1) The hydration process of the binary system at low temperature could be divided into three stages, namely, nucleation and growth (NG), interactions at phase boundaries (I), and diffusion (D);
(2) The *n* values for the binary system varied in the range of 1.2 to 1.6, indicating that the hydration of the binary system at low temperature was a complex elementary reaction;
(3) Silica fume can reduce the total heat at the later stage of the hydration and the reaction order *n*, but increase the heat flow at the accelerating stage and the hydration rate constant *K*. Low temperature prolonged the hydration induction period, decreased and delayed the secondary exothermic peak, as well as reduced the *n* and *K* value.

## Figures and Tables

**Table 1:** Chemical composition of Portland cement (wt%).

CaO	SiO_2_	Al_2_O_3_	Fe_2_O_3_	MgO	SO_3_	NaO_2_	Loss	Others
63.66	21.26	4.50	2.80	1.66	2.58	0.18	2.66	0.7

**Table 2:** Chemical composition of silica fume (wt%).

SiO_2_	Al_2_O_3_	Fe_2_O_3_	CaO	SO_3_	MgO	K_2_O	Loss	Others
86.62	0.51	1.52	1.02	0.58	2.77	2.03	3.88	1.07

**Table 3:** Mix proportion of the specimens.

No.	Cement (g)	Silica Fume (g)	Water (g)	Sodium Nitrite (g)	Sand (g)
A	450	0	189	0	1350
B	441	9	189	0	1350
C	427.5	22.5	189	0	1350
D	414	36	189	0	1350
E	405	45	189	0	1350
F	396	54	189	0	1350
G	450	0	189	38.25	1350
H	441	9	189	38.25	1350
I	427.5	22.5	189	38.25	1350
J	414	36	189	38.25	1350
K	405	45	189	38.25	1350
L	396	54	189	38.25	1350

**Table 4:** Hydration kinetic parameters of the composite cementitious material with varied silica fume contents.

	n	NG		I		D		α_1_	α_2_
		K_NG_	sd	K_I_	sd	K_D_	sd		
0	1.57	1.60×10^−2^	2.33×10^−3^	3.52×10^−3^	1.32×10^−2^	7.28×10^−4^	1.05×10^−3^	0.1	0.28
2%	1.52	1.61×10^−2^	3.52×10^−3^	3.69×10^−3^	8.96×10^−3^	7.59×10^−4^	8.37×10^−4^	0.1	0.28
5%	1.5	1.70×10^−2^	1.32×10^−3^	4.05×10^−3^	8.57×10^−3^	8.26×10^−4^	6.34×10^−4^	0.12	0.28
8%	1.49	1.79×10^−2^	4.68×10^−3^	4.22×10^−3^	7.50×10^−3^	9.67×10^−4^	4.57×10^−4^	0.11	0.31
10%	1.48	1.83×10^−2^	3.00×10^−3^	4.35×10^−3^	7.02×10^−3^	1.00×10^−3^	1.47×10^−3^	0.11	0.31
12%	1.48	1.85×10^−2^	3.58×10^−3^	4.42×10^−3^	7.84×10^−3^	1.02×10^−3^	1.39×10^−3^	0.12	0.31

**Table 5:** Hydration kinetic parameters of the composite cementitious system at different temperatures.

		n	NG		I		D		α_1_	α_2_
			K_NG_	sd	K_I_	sd	K_D_	sd		
Pure cement	5℃	1.57	1.60×10^−2^	2.08×10^−3^	3.52×10^−3^	1.20×10^−2^	7.28×10^−4^	1.79×10^−3^	0.10	0.28
	0℃	1.36	9.27×10^−3^	7.70×10^−3^	2.39×10^−3^	7.70×10^−3^	4.50×10^−4^	2.30×10^−3^	0.13	0.26
	−5℃	1.32	7.02×10^−3^	6.92×10^−4^	1.66×10^−3^	3.75×10^−3^	3.14×10^−4^	5.29×10^−3^	0.08	0.26
	−10℃	1.29	5.89×10^−3^	1.28×10^−3^	1.43×10^−3^	1.05×10^−2^	3.05×10^−4^	1.44×10^−2^	0.09	0.29
8% silica fume	5℃	1.49	1.79×10^−2^	2.48×10^−3^	4.22×10^−3^	3.98×10^−3^	9.67×10^−4^	2.26×10^−3^	0.11	0.31
	0℃	1.32	1.13×10^−2^	3.15×10^−3^	2.81×10^−3^	4.79×10^−3^	6.66×10^−4^	4.09×10^−4^	0.11	0.32
	−5℃	1.29	8.29×10^−3^	2.10×10^−3^	1.97×10^−3^	3.69×10^−4^	4.57×10^−4^	3.14×10^−3^	0.08	0.31
	−10℃	1.25	6.87×10^−3^	2.64×10^−3^	1.79×10^−3^	7.49×10^−3^	4.41×10^−4^	1.05×10^−2^	0.11	0.33
